# Opinions on Ketogenic Diets Among Students and Academic Teachers at the University of Pécs, Hungary: A Cross-Sectional Survey

**DOI:** 10.3390/nu17213327

**Published:** 2025-10-22

**Authors:** Nicole Hunter, János Girán, Gergely Márovics, Károly Berényi, Balázs Németh, Katalin Szendi

**Affiliations:** Department of Public Health Medicine, Medical School, University of Pécs, 7624 Pécs, Hungary

**Keywords:** ketogenic diet, knowledge, perceptions, university students, academic staff, cross-sectional survey, nutrition surveys, misinformation

## Abstract

**Background:** The ketogenic diet (KD) is one of the most widely followed dietary approaches worldwide, frequently promoted in popular media for weight loss and chronic disease management, although it also has established therapeutic applications in clinical medicine. However, our previous reviews have shown that existing clinical studies and meta-analyses on KD suffer from serious methodological limitations, raising concerns about their reliability. Considering this, the present study aimed to assess knowledge and opinions on KD among university students and academic staff. **Methods:** Cross-sectional, self-developed online questionnaire was distributed to 23,330 students and academic teachers at the University of Pécs, Hungary. Data was collected in October 2024. A total of 891 responses were included (710 students, 123 academic staff). Knowledge scores were calculated (maximum 17 points, including penalties for incorrect answers) and data were analyzed using descriptive statistics, ANOVA, chi-square tests, Pearson correlation, and logistic regression. **Results:** Only 7.3% of students and 13.5% of staff achieved ≥60% of the maximum knowledge score. Health-related faculties did not consistently outperform non–health-related ones; in fact, some non-health-related faculties achieved the highest mean scores. Completion of nutrition-related courses and reliance on PubMed were associated with higher knowledge, while current KD adherence among staff was negatively associated. Most participants (over 65%) were uncertain about the reliability of KD research. **Conclusions:** Knowledge of KD among both students and staff was limited, highlighting susceptibility to misinformation. Critical appraisal skills and reliable nutrition education are urgently needed at the population level to support disease prevention and to counterbalance misleading claims about KD.

## 1. Introduction

The ketogenic diet (KD) has recently gained notable popularity [1], both as a weight-loss strategy and as a potential therapeutic approach for several medical conditions [2,3,4,5,6,7,8,9]. The diet is characterized by a high fat content, and a very low carbohydrate intake [10]. Previous studies [11] have demonstrated that achieving nutritional ketosis (which is defined as having a blood ketone concentration of 0.5–3.0 mmol/L) was most effective when consuming the ketogenic diet macronutrient distribution recommended by Harvard University. The macronutrient distribution consisted of approximately 70–80% of one’s total energy from fat, 5–10% from carbohydrates, and 10–20% from protein. Harvard also stated that one can achieve nutritional ketosis by consuming even more fat and even less carbohydrates (than outlined above) as long as the person is consuming the standard ~2000 kcal daily [10]. However, these findings were obtained only when participants consumed meals prepared exclusively by the researchers [11].

Several meta-analyses [12,13,14,15,16,17,18,19,20,21,22,23,24,25,26,27] have reported beneficial associations between the ketogenic diet and outcomes such as body weight, blood lipids, and glycemic parameters. Based on these findings, the ketogenic diet is often recommended as an effective diet [28]. Given that meta-analyses comprise evidence from clinical studies, they are typically regarded as the most reliable source of information. However, in the case of ketogenic diet research, the conclusion drawn by these meta-analyses should not be immediately accepted at face value. In our previous work [28], we reviewed 28 meta-analyses on ketogenic diets and identified six major confounding factors (e.g., sustained adherence to the ketogenic diet could not be achieved, or lower caloric intake in the ketogenic diet group than in the control group) that undermine the validity of their conclusions. To further investigate the claims of these meta-analyses, we conducted a systematic literature review of clinical trials examining ketogenic diets in individuals with obesity or diabetes [11]. As a result, we identified three additional critical sources of bias (deviations from the planned macronutrient ratios, lack of verification of nutritional ketosis, caloric restriction or starvation effects) that compromised the reliability of the reported outcomes. Existing clinical studies on ketogenic diets are often limited by self-reported adherence, heterogeneous definitions, and inconsistent follow-up durations [29]. The combined use of meta-analyses and the serious confounding factors embedded within them created a misleadingly strong case in favor of the ketogenic diet despite claims by other fields of medicine that this dietary approach may lead to harmful effects [28].

Consequently, even professionals and/or the public that are familiar with the scientific literature on ketogenic diets may not be able to apply their knowledge without potential adverse consequences. Therefore, in the present study, we aimed to evaluate the knowledge and perceptions of both academic teachers and students at the University of Pécs regarding the effects of ketogenic diets via survey. Furthermore, we sought to determine whether participants were aware that scientific evidence can sometimes be misleading.

Our hypothesis was that a majority of respondents (who lacked formal training in nutrition science, physiology, or pathophysiology) would not critically question the findings of scientific studies on ketogenic diets, even if they were familiar with the general principles of healthy eating or the findings of recent research. We anticipated that academic teachers, upper-year medical or pharmacy students (years 4–6) at the Faculty of Medicine and Faculty of Pharmacy, and upper-year students in the dietetics program at the Faculty of Health Sciences, would provide more accurate responses. We assumed that participants from other faculties would be considered representative of the general population due to their limited prior training. Should our results deviate from this expectation, it would further underscore the importance of promoting appropriate attitudes toward nutrition both across university faculties, and within the broader population.

Several other questionnaire surveys [30,31,32,33,34,35,36,37,38] have also examined respondents’ familiarity with the ketogenic diet focusing on university students (with or without a background in health sciences), and members of the general public’s knowledge and perception of the diet. These questionnaires generally addressed factual aspects of the ketogenic diet that are well established within scientific circles. However, these questionnaires did not consider the reliability of ketogenic diet research, participants’ awareness of these methodological issues, or other prevalent misconceptions.

We developed our questionnaire specifically to explore the knowledge and perceptions of both academic teachers and students regarding the ketogenic diet, despite the recognized limitations and uncertainties within the scientific literature [11,28]. Based on the current body of evidence in medicine and health sciences, the correct answers to our questionnaire can only be inferred in some cases, or at best assumed with a high degree of certainty. Furthermore, our questionnaire was meant to address gaps in clinical research, common misconceptions, and errors in interpretation. To our knowledge, such an approach has not previously been undertaken in Hungary or elsewhere.

Understanding how future healthcare professionals and academic communities perceive and interpret information about ketogenic diets is crucial, as these groups play a key role in shaping public opinion, patient counselling, and evidence-based dietary recommendations. Assessing their knowledge and misconceptions can reveal critical educational gaps that may ultimately affect clinical practice and public health communication. Therefore, identifying the extent and accuracy of their understanding provides valuable insights into how misinformation about ketogenic diets might propagate within both professional and lay contexts.

## 2. Materials and Methods

### 2.1. Research Design

A cross-sectional study was conducted using a voluntary, self-selection, non-probability sampling method among undergraduate students as well as academic staff at the University of Pécs, Hungary. Data was collected with a self-developed novel questionnaire specifically designed for this research. The study was conducted in accordance with the Declaration of Helsinki and was approved by the Scientific and Research Ethics Committee of the Hungarian Medical Research Council (ETT TUKEB) (approval number: BM/15050-3/2024, date of approval: 3 August 2024).

### 2.2. Sample

A total of 23,330 invitation emails were sent to students and academic staff, and the surveys were distributed via Google Forms. Data collection took place in October 2024. The target population included students and academic staff from all ten faculties of the University of Pécs (Medical School; Faculty of Pharmacy; Faculty of Health Sciences; Faculty of Law; Faculty of Humanities and Social Sciences; Faculty of Business and Economics; Faculty of Cultural Sciences, Education and Regional Development; Faculty of Engineering and Information Technology; Faculty of Music and Visual Arts; Faculty of Sciences), with questionnaires available in both Hungarian and English. The first three faculties provide education in health sciences, which also includes training on healthy nutrition. Respondents from the remaining seven faculties can be considered representative of the general population in terms of their knowledge related to different types of nutrition.

Inclusion criteria required participants to be adults (≥18 years), either students or academic staff of the University of Pécs, and to have completed the entire questionnaire. Exclusion criteria included incomplete or invalid survey responses, as well as a lack of affiliation (active student or academic staff member) with the University of Pécs.

### 2.3. Procedures

Authorization from the Rector was obtained to recruit voluntary participants. Student and staff email addresses were provided by the University’s Provost Office. The invitation email included a hyperlink directing recipients to the informed consent form and the questionnaire.

Participation in the study was voluntary, was not incentivized, and informed consent was obtained electronically before respondents could access the questionnaire. The consent form outlined the aims of the study, provided the name and contact information of the principal investigator, and emphasized that participants could withdraw from the research at any time without any negative consequences. The invitation email also explained the purpose of the survey (to explore the perceptions of students and academic staff at the University of Pécs regarding the ketogenic diet) and highlighted the value of their contribution. Furthermore, participants were assured that no personally identifiable data (e.g., names or IP addresses) would be collected, and that all responses would remain confidential. To enhance the response rate, two rounds of invitations were distributed. The first round of emails was sent to all eligible students and staff, followed by a reminder email two weeks later. There were no reminders beyond the two emails.

In compliance with the General Data Protection Regulation (GDPR, Regulation (EU) 2016/679) and national data protection laws, only data strictly necessary to achieve the research objectives were collected. All responses were anonymized, handled with full confidentiality, and processed exclusively for scientific purposes. Under no circumstances was data disclosed to unauthorized third parties. Participants’ rights to privacy, voluntary participation, and withdrawal without consequence were fully respected throughout the study.

### 2.4. Measurement Instruments

We employed an online questionnaire developed by members of our research team to collect data for this cross-sectional study. To ensure participants’ willingness to respond and fully complete the questionnaire, primarily closed-ended questions were included. The survey was designed so that it could be completed within approximately 12 min. To avoid influencing responses, explanatory notes were provided only when necessary to offer technical guidance (e.g., indicating that multiple answers could be selected).

Prior to distribution, a pilot test was conducted to ensure clarity and face validity of the questionnaire, as well as to assess the time required for completion. For this phase, 10 students and 10 academic staff members were asked to complete the questionnaire and provide feedback on the wording of the items. Because the questionnaire items primarily assessed factual knowledge rather than latent psychological constructs, internal consistency measures such as Cronbach’s alpha or test–retest reliability were not applicable. Instead, face and content validity were ensured through pilot testing and expert review.

The final version of the questionnaire (Supplementary Material Table S1) consisted of three main sections: (1) socio-demographic variables, (2) general questions regarding ketogenic diets, and (3) questions assessing participants’ knowledge.

The first section of the questionnaire included 18 items addressing demographic characteristics of students and academic staff. Participants were asked to report their sex, age, nationality, academic role (student or staff), faculty affiliation (workplace or study program), year of study, whether they had taken a nutrition-related course, height, weight, gym attendance, and whether they had ever heard of the ketogenic diet. Respondents who answered “no” to the latter question were directed to the end of the survey. To account for potential misclassification due to increased lean body mass, participants who reported attending the gym five times or more per week and engaging primarily in weightlifting, strength-based, or machine-based exercises were reclassified as normal weight even if their BMI fell within the overweight or obese range.

The second section consisted of four items covering general aspects of ketogenic diets. Participants were asked whether they currently follow, had previously followed, or intended to follow a ketogenic diet for weight loss or health reasons. Additional questions addressed whether a healthcare professional had ever recommended a ketogenic diet to the respondents, and respondence’s perceptions of the reliability of scientific research on ketogenic diets.

The third section contained 10 knowledge-based questions designed to assess participants’ understanding of the ketogenic diet. Respondents were asked to identify the main characteristics of a ketogenic diet from a list of 16 statements, to list common carbohydrate sources, to define nutritional ketosis, to estimate caloric intake in a ketogenic diet, and to indicate what they considered the most important and effective causes of weight loss. Respondents also had to select what they thought was the typical macronutrient distribution of a ketogenic diet, and to identify which types of fatty acids are considered healthy, and in which food sources they can typically be found. Finally, participants were asked about sources they regarded as reliable for information on ketogenic diets.

Sample size estimation was performed using an online calculator (https://www.calculator.net/sample-size-calculator.html, 23 September 2025). Assuming a 95% confidence level, a 5% margin of error, and a population proportion of 50%, the minimum required sample size was calculated to be 385 participants to ensure adequate statistical power.

### 2.5. Statistical Analysis

Data was analysed using IBM SPSS Statistics software, version 26.0 (IBM Corp., Armonk, NY, USA). Descriptive statistics (frequencies, percentages, means, and standard deviations) were calculated for demographic variables of students and academic staff. The normality of the data distribution was assessed using the Shapiro–Wilk test. Based on the results of the normality test, appropriate parametric or non-parametric tests were applied. Inferential statistical analyses included one-way analysis of variance (ANOVA), Pearson’s correlation, multivariate logistic regression, Mann–Whitney U test, and chi-square test. ANOVA was used to compare knowledge scores across faculties and study programs, followed by Dunnett’s T3 post hoc test where applicable. The Mann–Whitney U test was applied for comparisons between two independent groups (e.g., male vs. female participants). Pearson’s correlation was used to assess relationships between continuous variables (e.g., age and knowledge scores). Chi-square tests examined associations between categorical variables (e.g., adequate vs. inadequate knowledge and demographic characteristics). Finally, multivariable logistic regression models were constructed to identify independent predictors of awareness, adherence, and adequate knowledge scores among students and academic staff. Statistical significance was set at *p* < 0.05.

## 3. Results

Of the 23,330 invitation emails distributed, a total of 891 completed questionnaires were returned, including 661 in Hungarian and 230 in English. Among the respondents, 710 were students and 123 were academic staff. The response rate was 3.32% for students and 6.29% for staff. For institutional reasons, further breakdown of the sampling frame was not available. In addition, 57 employees not involved in teaching also completed the questionnaire; however, their responses were excluded from the analysis. For administrative reasons, the questionnaire was not distributed to physicians working at the university clinics who are also involved in teaching activities. Consequently, the views of clinical practitioners were not represented in this survey.

Table 1 presents the demographic characteristics of the students and academic staff who participated in the study. Information on the nationality of students and academic staff is provided in Supplementary Material Table S2.

The majority of respondents were female. Most of the completed questionnaires originated from the Medical School, representing 33.2% of students and 51.6% of academic staff respondents. Overall, 44.8% of the students were enrolled in non-health-related programs. At the time of the survey’s administration, the majority of students (*n* = 505; 71.1%) and academic staff (*n* = 111; 90.2%) reported having heard of the ketogenic diet. Participants who had not previously heard of the ketogenic diet were excluded from further analyses, as they were unable to complete the subsequent sections of the questionnaire.

### 3.1. Knowledge Score

A knowledge score was created based on participants’ responses to the items assessing familiarity with the ketogenic diet. The maximum achievable score was 17 points. Up to six penalty points could be assigned for incorrect answers to basic questions that reflected easily attainable knowledge. Incorrect responses that represented misleading information due to confounding factors in the scientific literature were scored as zero. The detailed scoring method is presented in Table 2.

**Figure 1:**
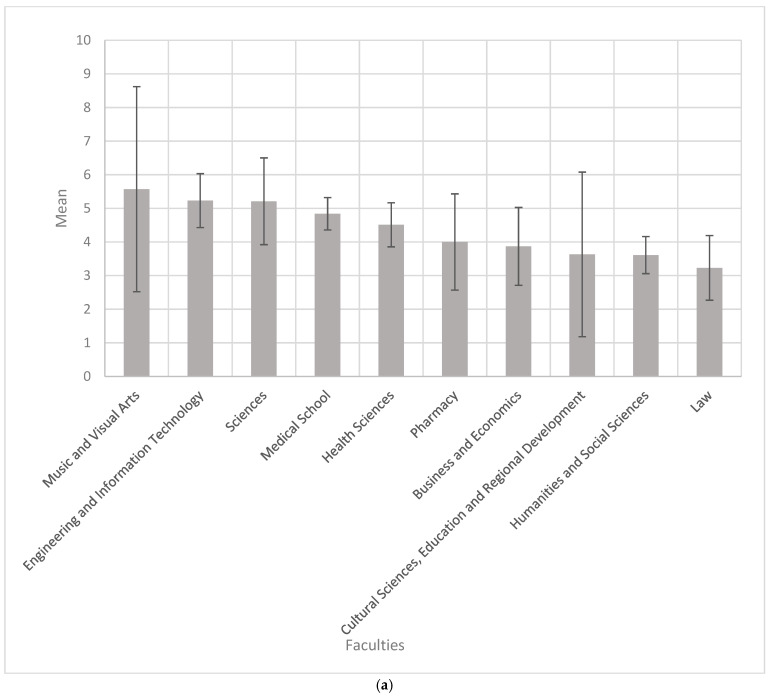

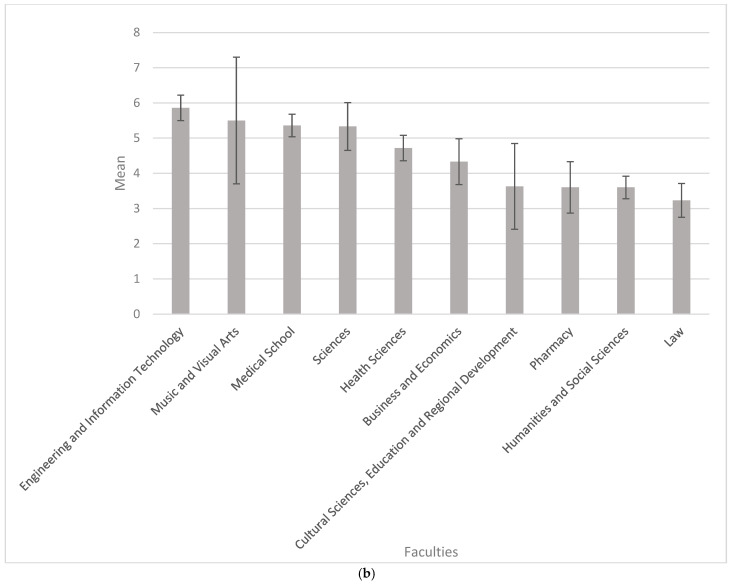

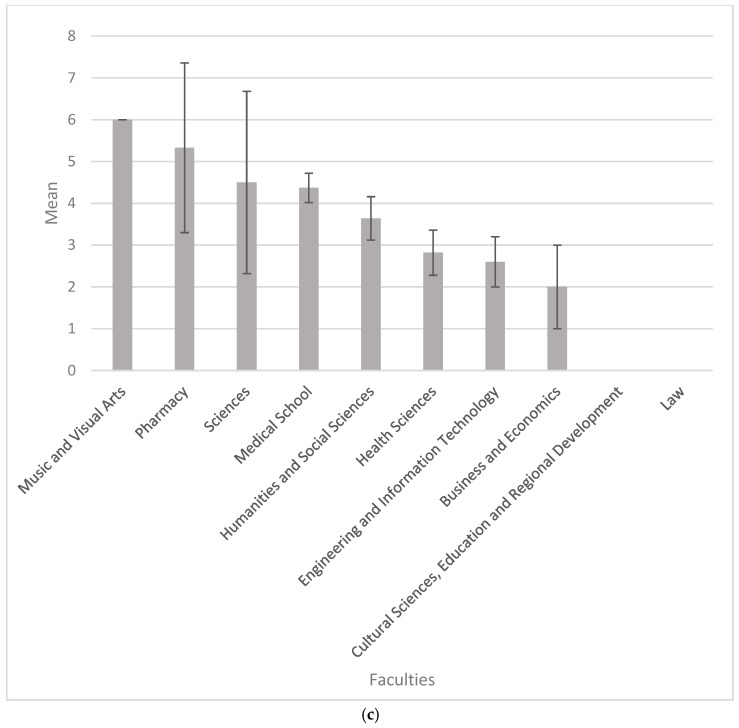
(**a**) Mean knowledge scores (±2 SEM) of students studying in the Hungarian and English program across faculties. (**b**). Mean knowledge scores (±2 SEM) of students studying in the Hungarian program across faculties. (**c**) Mean knowledge scores (±2 SEM) of students studying in the English program across faculties.

No significant difference in knowledge scores was observed between male and female participants, either among students (Mann–Whitney U test, *p* = 0.892) or academic staff (*p* = 0.327). When Hungarian- and English-speaking students were analyzed together, knowledge scores in most cases did not deviate substantially from a normal distribution; therefore, a one-way ANOVA with Dunnett’s T3 post hoc test was performed. The ANOVA indicated a significant difference between faculties (*p* = 0.017). According to Dunnett’s T3 test, students from the Medical School achieved significantly higher scores compared to those from the Faculty of Humanities and Social Sciences (*p* = 0.041), with a mean difference of 1.231 points. Although students from the Faculty of Music and Visual Arts obtained the highest mean score, the differences compared with other faculties were not statistically significant (Figure 1a).

Among Hungarian students, the ANOVA indicated a significant difference in knowledge scores between faculties (*p* = 0.001). Post hoc analysis with Dunnett’s T3 test revealed that students from the Medical School scored significantly higher than those from the Faculty of Law (mean difference = 2.131 points, *p* = 0.022) and the Faculty of Humanities and Social Sciences (mean difference = 1.753 points, *p* = 0.007). In addition, students from the Faculty of Engineering and Information Technology achieved significantly higher scores than those from the Faculty of Law (mean difference = 2.631 points, *p* = 0.003) and the Faculty of Humanities and Social Sciences (mean difference = 2.254 points, *p* = 0.001). No further significant differences between faculties were observed among Hungarian students. See Figure 1b.

Among English-program students, the ANOVA did not reveal any significant differences in knowledge scores between faculties (*p* = 0.384). See Figure 1c.

Among Hungarian- and English-program academic staff, the ANOVA did not show any significant differences in knowledge scores between faculties (*p* = 0.435).

The highest knowledge score was 15 out of 17 points, achieved by a Hungarian dietitian student from the Faculty of Health Sciences (MSc in Nutritional Sciences). The second-highest score was 14 points, also obtained by a student with the same background.

Among students, a higher knowledge score was weakly correlated with a lower number of incorrect answers. Pearson’s correlation analysis showed a correlation coefficient of r = 0.115 (*p* = 0.010).

A summary of the above-mentioned results is presented in Table 3.

We further examined which factors were associated with participants’ knowledge scores (e.g., sex, age, year of study, etc.). Students and staff who achieved at least 60% of the maximum possible knowledge score were classified as having “adequate knowledge,” while those scoring below 60% were categorized as having “inadequate knowledge.” Overall, 7.3% of students and 13.5% of staff reached the adequate knowledge threshold.

The following independent variables were assessed for both students and staff: sex, age, language of the questionnaire (Hungarian or English), completion of a nutrition-related course, BMI category adjusted for muscle mass, current adherence to a ketogenic diet, willingness to follow a ketogenic diet, and preferred sources of information on ketogenic diets. Year of study was also included as a variable for students.

Multivariable logistic regression showed that students who had completed a nutrition-related course were significantly more likely to achieve adequate knowledge (OR = 4.701, *p* = 0.001, 95% CI: 1.915–11.537). Identifying the Internet (OR = 2.615, *p* = 0.012, 95% CI: 1.233–5.545) or PubMed (OR = 4.475, *p* < 0.001, 95% CI: 1.957–10.231) as information sources also significantly increased the odds of achieving adequate knowledge.

Among staff, however, reporting current adherence to a ketogenic diet significantly decreased the likelihood of reaching adequate knowledge (OR = 0.430, *p* = 0.013, 95% CI: 0.221–0.834).

A summary of the above-mentioned results is presented in Table 4.

Nominal variables were analysed using chi-square tests with respect to whether participants achieved adequate knowledge scores (≥60%). Educational qualification, faculty, and field of study did not significantly influence the likelihood of students or staff reaching the threshold. Similarly, gym attendance, type of exercise performed (strength-based vs. aerobic training), engagement in physical activity outside of the gym, or current adherence to a ketogenic diet were not significantly associated with achieving adequate knowledge.

Among students, responses to the question “In your opinion, are the results of scientific research on ketogenic diets reliable?” were significantly associated with knowledge scores (*p* = 0.004). Of those who considered the research unreliable, 17.8% achieved adequate knowledge, compared with 10.3% of those who considered the research reliable, and only 5.1% of those who reported being unable to judge.

Among academic staff, no significant association was observed for this question (*p* = 0.060).

Among the questions contributing to the knowledge score, one of the most important and informative items was: “Please list as many types of foods that typically contain carbohydrates as you can.” The highest scores were awarded to participants who identified both vegetables and fruits as major carbohydrate sources. This was correctly reported by 27.3% of respondents who completed the Hungarian-language questionnaire and 18.6% of those who completed the English-language version.

Another key item asked: “In your opinion, what characterizes nutritional ketosis?” Only 5.8% of all participants selected all three correct answers (students: 5.9%, staff: 5.4%, Medical School students: 8.8%).

We also assessed a fundamental principle which closely aligned with the law of conservation of energy via the question: “In your opinion, is a sufficiently low-calorie intake enough for weight loss?” Overall, 49.2% of participants responded correctly (“yes”).

Another essential question was: “When following a ketogenic diet, what can one eat?” to which 68% of participants provided the correct response (students: 67.5%, staff: 70.3%).

Finally, two additional key items, which also formed part of the knowledge score, addressed dietary fats: “In your opinion, are animal-derived fats or plant-derived fats healthier?” and “Which types of fatty acids are mainly found in animal-derived foods?” Since fat is the predominant macronutrient consumed in ketogenic diets, these items were extremely relevant. Only 28.2% of participants correctly identified plant-derived fats as the healthier option, while 53.1% correctly recognized that animal-derived fats are predominantly composed of saturated fatty acids.

The descriptive results of key questionnaire items and their response distributions are summarized in Table 5.

### 3.2. Question 1: “Have You Heard of Ketogenic Diet(s) Before?”

We further applied multivariable logistic regression to examine which factors influenced the likelihood of having heard of the ketogenic diet (Question 1). For students, the independent variables included sex, age, year of study, language of the questionnaire (Hungarian or English), completion of a nutrition-related course, and BMI category adjusted for muscle mass.

Among students, females were significantly more likely to have heard of the ketogenic diet (OR = 1.456, *p* = 0.043, 95% CI: 1.012–2.097). Increasing age was also associated with a higher likelihood of awareness (OR = 1.030, *p* = 0.007, 95% CI: 1.008–1.053), as was advancing year of study (OR = 1.201, *p* = 0.001, 95% CI: 1.082–1.335). Students who completed the English-language version of the questionnaire were significantly less likely to have heard of the ketogenic diet compared to those who completed the Hungarian version (OR = 0.558, *p* = 0.002, 95% CI: 0.389–0.800). Students without prior coursework in nutrition were substantially less likely to have heard of the ketogenic diet (OR = 0.208, *p* = 0.003, 95% CI: 0.072–0.596).

For academic staff, the independent variables analysed included sex, age, language of the questionnaire, and BMI category adjusted for muscle mass. Only one significant association was identified: staff who completed the English-language questionnaire were less likely to have heard of the ketogenic diet compared to those who completed the Hungarian version (OR = 0.046, *p* = 0.015, 95% CI: 0.004–0.551).

The summarized results of the multivariable logistic regression analysis for Question 1 are presented in Table 6.

### 3.3. Question 2: “Are You Currently Following a Ketogenic Diet?”

Next, we applied multivariable logistic regression to examine which factors influenced the likelihood of responding Question 2. Independent variables analysed for both students and staff included sex, age, year of study (students only), language of the questionnaire (Hungarian or English), completion of a nutrition-related course, whether a healthcare professional had recommended the ketogenic diet previously, and BMI category adjusted for muscle mass.

Among students, receiving a recommendation from a healthcare professional significantly increased the likelihood of following a ketogenic diet (OR = 4.375, *p* = 0.001, 95% CI: 1.874–10.212). Older age was also associated with a significantly higher likelihood of currently or previously following the diet (OR = 1.044, *p* < 0.001, 95% CI: 1.023–1.066). No other variables were significantly associated with ketogenic diet adherence among students.

For academic staff, none of the examined variables showed a significant association with current or past adherence.

Of all respondents (*n* = 616), 17 (2.8%) reported currently following a ketogenic diet, while 99 (16.1%) had tried it previously (students: 2.6%/16.2%; staff: 3.6%/15.3%, respectively).

The summarized results of the multivariable logistic regression analysis for Question 2 are presented in Table 7.

### 3.4. Question 3: “Would You Follow a Ketogenic Diet for the Purpose of Losing Weight, or Being “More Healthy”?”

For the Question 3 the following independent variables were analysed: sex, age, year of study (students only), language of the questionnaire (Hungarian or English), completion of a nutrition-related course, and BMI category adjusted for muscle mass.

Among students, females were significantly less likely to report willingness to follow a ketogenic diet for weight loss or health reasons (OR = 0.549, *p* = 0.005, 95% CI: 0.362–0.832). In contrast, older age was associated with a significantly greater likelihood of choosing the diet for these purposes (OR = 1.036, *p* < 0.001, 95% CI: 1.017–1.057).

No significant associations were found among academic staff.

Overall, 30.8% of all participants indicated that they would consider following a ketogenic diet for weight loss or health-related reasons.

The summarized results of the multivariable logistic regression analysis for Question 3 are presented in Table 8.

### 3.5. Question 4: “Has a Healthcare Professional Ever Suggested You Follow a Ketogenic Diet?”

In response to the Question 4 the following results were obtained. Among students, 25 participants (5.0%) reported having received such a recommendation. The proportion was 5.2% among Hungarian students and 4.3% among English-track students, with no significant difference between the two groups (*p* = 0.861).

Chi-square analysis showed that 3.7% of non-overweight students versus 7.9% of overweight students reported receiving a recommendation from a healthcare professional (*p* = 0.044), indicating that overweight students were significantly more likely to be advised to follow the ketogenic diet. Furthermore, among those who had received such a recommendation, 52% had previously tried or were currently following the diet, compared with only 17.1% of those who had not received a recommendation (*p* < 0.001).

No academic staff member reported ever having been advised by a healthcare professional to follow a ketogenic diet.

The summarized chi-square results for Question 4 are presented in Table 9.

### 3.6. Question 5: “In Your Opinion, Are the Results of Scientific Research Done on Ketogenic Diets Reliable?”

**Figure 2:**
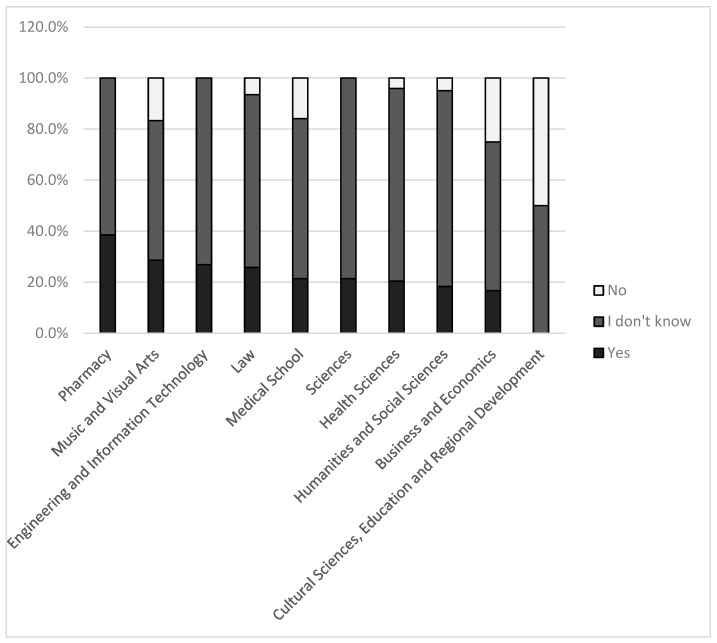
Students’ perceptions of the reliability of ketogenic diet research by faculty.

Chi-square tests were also conducted to examine whether responses to Question 5 were significantly influenced by the following independent variables: faculty, field of study, year of study (students only), completion of a nutrition-related course, current or past adherence to a ketogenic diet, willingness to follow a ketogenic diet, and preferred sources of information on ketogenic diets. The distribution of responses regarding the reliability of scientific research differed significantly across faculties (*p* = 0.035). The percentage distribution of responses by faculty is presented in Figure 2. Notably, 38.5% of students from the Faculty of Pharmacy considered the results of scientific research to be reliable.

**Figure 3:**
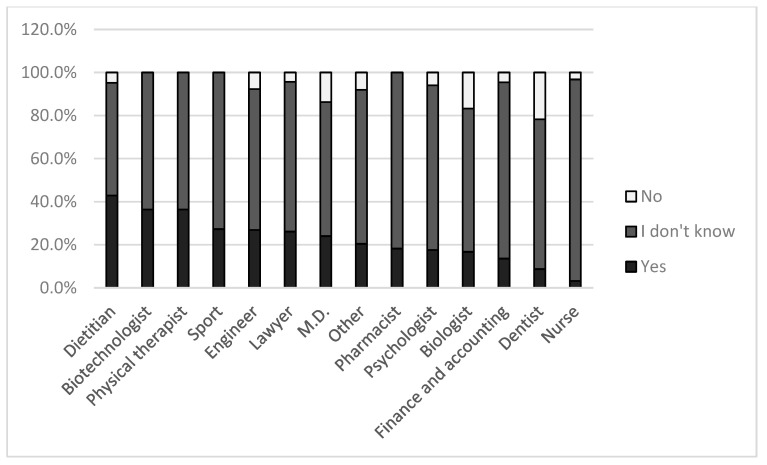
Students’ perceptions of the reliability of ketogenic diet research by field of study.

Across different fields of study, the distribution of responses regarding the reliability of scientific research on ketogenic diets differed significantly (*p* = 0.020). The percentage distribution of responses by field is presented in Figure 3. Notably, a strikingly high proportion of dietetics students (42.9%) considered the results of ketogenic diet research to be reliable (*p* = 0.020).

No significant differences were observed by year of study.

Among students, the distribution of responses to the question “In your opinion, are the results of scientific research on ketogenic diets reliable?” differed significantly depending on whether they reported “Are you currently following a ketogenic diet?” (*p* = 0.005). Of those currently or previously following the diet, 31.6% considered scientific research reliable, 3.2% did not, and the remainder selected “I don’t know.” Among students who had never followed a ketogenic diet, 18.8% regarded the research as reliable, 10.2% as unreliable, and the rest chose “I don’t know.”

Similarly, student responses to “Would you follow a ketogenic diet for the purpose of losing weight or being ‘more healthy’?” were also significantly associated with perceived reliability of scientific research (*p* < 0.001). Among those who indicated willingness to follow the diet, 29.4% considered the research reliable, 3.1% unreliable, and 67.5% selected “I don’t know.” In contrast, among students unwilling to follow the diet, only 17.3% viewed the research as reliable, 11.7% as unreliable, and 71.1% responded “I don’t know.”

For academic staff, responses to the same question also differed significantly (*p* = 0.001). Among staff willing to follow the ketogenic diet, 37% judged the research reliable, 63% selected “I don’t know,” and none indicated that they considered the research unreliable. In contrast, among those unwilling to follow the diet, only 10.7% regarded the research as reliable, 14.3% as unreliable, and 75% responded “I don’t know.”

The summarized chi-square results for Question 5 are presented in Table 10.

### 3.7. Question 6: “If You Wanted to Learn More About Ketogenic Diets, Which of These Resources Would You Use?”

**Figure 4:**
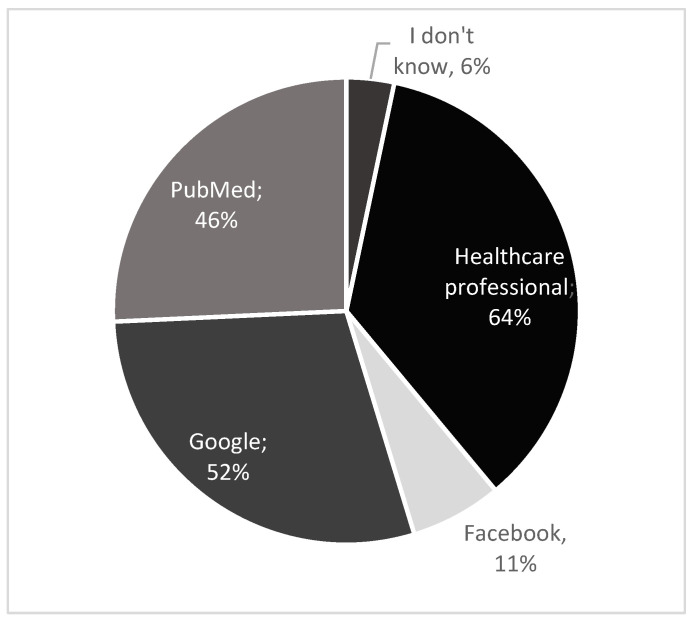
Students’ preferred resources for obtaining information on ketogenic diets.

**Figure 5:**
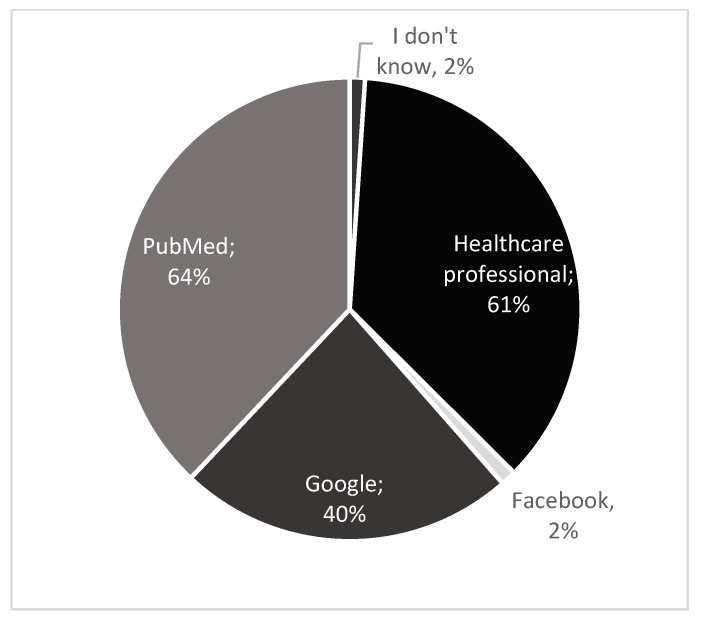
Academic staff’s preferred resources for obtaining information on ketogenic diets.

Among students, the most frequently chosen source was healthcare professionals, whereas academic staff mostly reported PubMed as their primary source of information (Figure 4 and Figure 5). The question was multiple choice. The percentages shown in the pie charts represent the proportion of respondents who selected each answer option. The charts also illustrate the relative frequency of the individual response options.

## 4. Discussion

To the best of our knowledge, this is the first questionnaire-based study on ketogenic diets that specifically addresses gaps in knowledge and application arising from current clinical research, as well as misconceptions and common errors. Previous surveys on ketogenic diets did not include such aspects [30,31,32,33,34,35,36,37,38]. Instead, they often assessed participants’ knowledge of facts that, due to methodological limitations of the underlying studies, cannot be considered reliably known. Our earlier critical review [11] and our review of existing meta-analyses [28] clearly demonstrated that within the research fields of obesity, and diabetes, neither individual clinical trials, nor meta-analyses provided reliable evidence on the effects of ketogenic diets. Our primary concern is that in many published clinical studies, the dietary interventions labelled as “ketogenic diets” were unlikely to induce nutritional ketosis in participants which makes sufficiently frequent ketone body testing critical for accurate adherence assessment [39]. Furthermore, in most cases, the caloric intake of the ketogenic diet groups was substantially lower than that of the control groups. This confounding factor makes it impossible to determine whether weight loss and improvements in blood parameters were attributable to the ketogenic diet intervention itself, or simply to a reduced energy intake.

Our questionnaire was self-developed, whereas most existing surveys either did not report the source of their instrument or referenced a source that itself lacked a clear origin [30,31,32,34,36,37,38]. Three published surveys included more participants than our study (*n* = 891). One collected data from 17 countries [31] (1292 participants), another study collected data from medical and non-medical students as well as the general population in Saudi Arabia [32] (1071 participants), although no information was provided on the number of universities included or how the general population was recruited. A third survey [30] was conducted at Kent State University (USA) with 1131 students across various majors, years of study, and enrollment status.

In our sample, 44.8% of students were enrolled in non-healthcare related specialties. Similarly, in the Saudi Arabian study by Alshaikh et al. [34], 64.6% of student respondents were in non-healthcare related programs. In our survey, the majority of students (*n* = 505; 71.1%) and academic staff (*n* = 111; 90.2%) reported being familiar with ketogenic diets at the time of data collection. This was consistent with Alshaikh et al. [34], where the majority of students (84.8%) also reported awareness of ketogenic diets.

The following section addresses whether our initial hypothesis was confirmed.

### 4.1. Knowledge Score

As shown in Figure 1a, which presents the mean knowledge scores of students enrolled in Hungarian- and English-language programs, our initial hypothesis (students from healthcare related faculties would demonstrate greater awareness of the effects and characteristics of ketogenic diets) was not confirmed. Surprisingly, students from the Faculty of Music and Visual Arts achieved the highest average knowledge scores among all ten faculties of the University of Pécs, even though they are unlikely to possess formal background knowledge in nutrition, physiology, or pathophysiology. However, these differences were not statistically significant compared to other faculties. The only significant difference observed was between the Medical School and the Faculty of Humanities and Social Sciences, with Medical School students achieving higher scores.

When considering only Hungarian students, Medical School students performed significantly better than students from the Faculty of Law and the Faculty of Humanities and Social Sciences. This finding is partially consistent with our initial hypothesis.

The highest individual knowledge score (15 out of 17) was achieved by a dietetics student in the Hungarian program, which also supports our hypothesis, as dietetics students focus most directly on nutrition science within their curriculum.

Interestingly, students with higher overall knowledge scores also accumulated penalty points corresponding to incorrect answers in the more severe category. This association may reflect the influence of unreliable scientific literature currently available on the effects of ketogenic diets.

It is discouraging that only 7.3% of students achieved at least 60% of the maximum possible knowledge score. Among academic staff, the proportion was somewhat higher (13.5%) yet still cannot be considered satisfactory.

Hussain et al. [38], who investigated medical university students (public health program students) in London using a questionnaire on the utilization, knowledge, and perception of the ketogenic diet, reported that participants demonstrated limited knowledge of the ketogenic diet. Similar to our study, medical students did not achieve the highest knowledge scores. However, it should be noted that our questionnaire assessed knowledge using items that differed from those in most other surveys. Alhassani et al. [32], who examined Saudi adults, medical and non-medical students, and the general population, found that students had significantly lower knowledge scores compared to non-students, while medical majors scored higher than non-medical majors. In our study, non-healthcare related faculties were intended to represent the general population, yet our findings did not indicate superior performance among healthcare related faculties. Similarly, D’Agostino et al. [30] reported significant differences in ketogenic diet knowledge between healthcare related and non-healthcare related majors (*p* = 0.018), as well as between diet followers and non-followers. Alhaj et al. [31] rated the knowledge of Arab adults from 17 countries as “good,” but it is important to note that all participants in that study were keto diet followers. Altamimi et al. [35] observed that students enrolled in Medical and Health Sciences faculties demonstrated low knowledge of ketogenic diets. Butt et al. [36] reported that medical students were able to correctly answer the questions posed, although no comparisons were made with other faculties or the general population.

With all this taken into consideration, our results suggest that students in healthcare related majors are not necessarily the most knowledgeable about ketogenic diets, which is in line with the findings of some of the previous survey-based studies. Since ketogenic diets are often portrayed as fad diets in the popular domain, they may receive limited formal attention in healthcare-related university curricula. Conversely, members of the general population (which includes individuals whose interests in ketogenic diets are more prevalent) may independently seek out information, which could explain their higher levels of awareness in some studies. This finding is in contradiction with our original hypothesis.

The following factors were found to be significantly associated with participants’ knowledge scores: (i) completion of any nutrition-related training or courses; (ii) identifying the Internet or PubMed as a primary source of information; (iii) current adherence to a ketogenic diet; and (iv) responses to the question “In your opinion, are the results of scientific research on ketogenic diets reliable?”

(i–ii) Students who had completed a nutrition-related course and those who reported the Internet or PubMed as their preferred sources of information tended to achieve the minimum knowledge threshold of 60%. Notably, only 6.6% of students had completed such a nutrition-related course. In the study by Hussain et al. [38], most participants (public health program students) had attended nutrition-related courses at university (71.1%).
(iii) Among academic staff, current adherence to a ketogenic diet was associated with a lower likelihood of achieving the 60% knowledge threshold. By contrast, Altamimi et al. [35] reported the opposite among university students in Medical and Health Sciences Faculties, where ketogenic diet users scored significantly higher. It should be emphasized, however, that the knowledge scores generated by the two questionnaires are not directly comparable.
(iv) Among students, only 17.8% of those who considered the studies on ketogenic diets to be unreliable achieved adequate knowledge scores (≥60%). Strikingly, among those who regarded the studies as reliable, the proportion was even lower (10.3%). This may reflect that these students were influenced by misleading scientific findings at face value and responded to the questions accordingly. These results highlight the need for critical appraisal, even when interpreting information from scientific literature.

Among the knowledge score items, the question “Please list as many types of foods that typically contain carbohydrates as you can” deserves special attention, as this represents a critical issue among Hungarian medical students, other healthcare related students, and within the general population. In Hungary, fruits and vegetables are typically not recognized as carbohydrate sources even by medical students. In everyday language, “carbohydrate sources” are most often associated with pasta, sugar, chocolate, and pastries. This misconception is reflected in the dietary habits of the Hungarian population, whose fruit and vegetable consumption falls short of the WHO recommendation of at least 400 g per day [40], as shown in the OTÁP 2019 survey [41]. On a typical Hungarian restaurant’s menu, vegetables are typically only present in minimal amounts as garnish, while desserts typically consist of cakes or pastries rather than fruit. Moreover, a substantial proportion of fruit production is consumed in the form of pálinka (fruit brandy) or jam. Unfortunately, only a small fraction of respondents could answer this question correctly. Only 27.3% among those completing the Hungarian-language questionnaire, and 18.6% among those completing the English-language version.

One of the greatest methodological shortcomings of clinical trials on ketogenic diets is the failure to verify the presence of nutritional ketosis [11]. If a study fails to measure whether participants on a ketogenic diet are actually in nutritional ketosis during the intervention or not, the diet cannot truly be classified as ketogenic, since no measurement supports that claim. In our survey, only 5.8% of respondents correctly answered the question “In your opinion, what characterizes nutritional ketosis?” with Medical School students achieving 8.8%. This is particularly concerning, given that researchers who are currently, or are planning to conduct studies on ketogenic diets may not even intend to measure nutritional ketosis, making it unlikely that they will produce reliable scientific results.

Another important item assessing knowledge in our questionnaire was: “In your opinion, is a sufficiently low-calorie intake enough for weight loss?” A common misconception among laypeople, and sometimes even among healthcare professionals (often reinforced by non-scientific internet sources) is that being overweight or obese is primarily attributable to medications, genetic background, or hormonal imbalances rather than caloric intake. Similar misconceptions were reflected in many of the responses in our survey. While these risk factors may indeed increase the likelihood of obesity, they cannot be considered the sole cause. By contrast, excessive caloric intake (when energy intake exceeds expenditure) is sufficient on its own to induce obesity. This is a fundamental principle that should be understood regardless of educational background. Nevertheless, less than half of the respondents (49.2%) provided the correct answer. In the context of ketogenic diets, the relevant question was not whether patients would lose weight when consuming fewer calories than they expend (since this is true of any diet) but whether weight loss occurs under a norm caloric ketogenic diet (~2000 kcal) as well. This would represent the true distinguishing feature of a ketogenic diet: weight reduction without caloric restriction [11,28]. This item was part of a sequence of questions designed to test understanding of energy balance and common misconceptions in ketogenic diet research.

Another important question was: “When following a ketogenic diet, what can one eat?” Not only is the general public unfamiliar with the appropriate macronutrient ratios required for the diet, but even some researchers studying the effects of ketogenic diet [28]. A common misconception among the general public is that ketogenic diets primarily involve consuming large amounts of meat. However, excessive protein intake can promote gluconeogenesis, thereby reducing ketone body production and preventing the diet from being truly ketogenic [28]. In our survey, 68% of respondents provided the correct answer [10]. By contrast, in the study by Altamimi et al. [35], only 30% of respondents (students enrolled in Medical and Health Sciences Faculties) were aware of the correct macronutrient ratios.

Two further key questions contributing to the knowledge score were: “In your opinion, are animal-derived fats or plant-derived fats healthier?” and “Which types of fatty acids are mainly found in animal-derived foods?” Most clinical studies investigating the effects of ketogenic diets in obese or diabetic patients unfortunately recommend the consumption of animal-derived fats [11]. Yet it is a well-established fact, both in the medical community and increasingly among the general public, that animal-derived fats are detrimental to health, contributing to both cardiovascular disease [42] and cancer development [43]. This raises the question of why dietary interventions labelled as ketogenic diets in research trials predominantly prescribe animal fats as the major macronutrient.

In our survey, more than half of respondents (53.1%) correctly identified that animal-derived fats are primarily composed of saturated fatty acids. However, fewer than one-third (28.2%) recognized that plant-derived fats are healthier than animal fats. This limited level of awareness is concerning, as effective prevention of cardiovascular and oncological diseases cannot be achieved without broader public understanding of such fundamental nutrition concepts.

### 4.2. Question 1: “Have You Heard of Ketogenic Diet(s) Before?”

The following factors were significantly associated with participants’ awareness of ketogenic diet: (i) sex; (ii) age; (iii) higher year of study; (iv) language of the questionnaire (Hungarian vs. English); and (v) completion of a nutrition-related course.

(i) Among students, females were more likely to have heard of the ketogenic diet, which may reflect a generally greater interest in health, body image, and nutrition among women.
(ii–iii) Older students and those in higher years of study were more likely to have heard of ketogenic diets, which may reflect greater exposure to information during their studies.
(iv) Interestingly, both students and academic staff who completed the English-language version of the questionnaire were less likely to have heard of ketogenic diet compared with those who completed the Hungarian version, despite the fact that the educational background is the same in both languages.
(v) Completion of a relevant nutrition-related course clearly contributed to students’ awareness of the existence of ketogenic diets.

### 4.3. Question 2: “Are You Currently Following a Ketogenic Diet?”

The following factors were significantly associated with whether participants were currently following ketogenic diet: (i) recommendation by a healthcare professional and (ii) age.

(i) Students who had been advised by a healthcare professional to follow ketogenic diet were significantly more likely to do so. This is not necessarily a misjudgment on the healthcare professional’s part, as they most likely relied on scientific evidence; even though, as highlighted in our previous work, such evidence is misleading for the conditions we analyzed [11,28].
(ii) Increasing age was also associated with a greater likelihood of currently or previously following a ketogenic diet, which might be explained by age-related lifestyle or awareness patterns.

Among our respondents (*n* = 616), 17 (2.8%) reported currently following ketogenic diet, while 99 (16.1%) had previously tried it. In comparison, Altamimi et al. [35] reported that 7.5% of respondents were ketogenic diet users. In contrast, Hasan et al. [37] found that 48% of their participants were currently following a ketogenic diet, which most likely reflects volunteer bias, as their target population was the general population in Baghdad. Finally, in the study by Alhaj et al. [31], all 1292 adult participants recruited from 17 Arab countries were ketogenic diet followers.

### 4.4. Question 3: “Would You Follow a Ketogenic Diet for the Purpose of Losing Weight, or Being ‘More Healthy’?”

Two factors were significantly associated with participants’ willingness to follow a ketogenic diet for weight loss or health purposes: (i) sex and (ii) age.

(i) Among students, females were significantly less likely to report a willingness to follow ketogenic diet for these purposes. This pattern may be related to greater awareness of healthy lifestyle practices and a more cautious attitude toward dietary trends commonly perceived as fad diets among women.
(ii) With increasing age, students were significantly more likely to express a willingness to follow a ketogenic diet, which may reflect, as suggested by our earlier findings, a greater likelihood of prior exposure to information on ketogenic diets.

A positive response to this question likely indicates that participants have been influenced by misinformation and lack adequate training in nutritional science. In our study, 30.8% of participants stated they would follow a ketogenic diet for weight loss or health reasons. In comparison, Altamimi et al. [35] reported that, among 227 students enrolled in Medical and Health Sciences Faculties, only 12 followed a ketogenic diet for the purpose of weight loss.

### 4.5. Question 4: “Has a Healthcare Professional Ever Suggested You Follow a Ketogenic Diet?”

Two factors were significantly associated with whether participants had ever been advised by a healthcare professional to follow ketogenic diet: (i) BMI and (ii) current or past adherence to ketogenic diet.

(i) Overweight students were significantly more likely than those with normal BMI to have received such a recommendation. This finding is understandable given that scientific literature on ketogenic diet, despite its methodological shortcomings, often highlights positive effects in obesity-related contexts.
(ii) Among those who had been advised by a healthcare professional to follow ketogenic diet, 52% had either previously tried it, or were currently adhering to it. This is a regrettably high proportion. However, since only 5% of student participants overall reported ever having received such a recommendation, the absolute number of cases remains relatively low, which is an encouraging sign that such recommendations are not yet widespread.

Healthcare professionals working in this area should be aware of the physiological and pathophysiological processes that contraindicate the recommendation of ketogenic diets. Especially, given that there exist a number of other well-established diets that provide proven long-term health benefits without extreme macronutrient imbalances.

### 4.6. Question 5: “In Your Opinion, Are the Results of Scientific Research Done on Ketogenic Diets Reliable?”

Several factors were significantly associated with whether participants considered scientific research on ketogenic diet to be reliable: (i) faculty, (ii) field of study, (iii) current or past adherence to ketogenic diet, and (iv) willingness to follow ketogenic diet for weight loss or health purposes.

(i) Students from the Faculty of Pharmacy most frequently regarded ketogenic diet research as reliable (38.5%; Figure 2), followed by the Faculty of Music and Visual Arts and the Faculty of Engineering and Information Technology. While the high ranking of two non-healthcare related faculties is understandable, the relatively high confidence among pharmacy students was unexpected in light of our initial hypothesis. By contrast, students from the Faculty of Cultural Sciences, Education and Regional Development expressed the lowest level of trust in ketogenic diet research.
(ii) Dietetics students stood out, with 42.9% believing that ketogenic diet research findings are reliable. This may reflect that their training emphasizes the practical application of scientific results rather than critical appraisal. Medical students fell in the middle range, consistent with the fact that their education explicitly encourages critical thinking. Nursing students were the most skeptical; however, the majority indicated insufficient knowledge to make a judgment. In general, as illustrated in Figure 3, most students across faculties did not provide a definitive answer to this question.
(iii) Among students who were current or past ketogenic diet followers, 31.6% considered the research reliable, compared to 18.8% among those who had never followed KD. Nevertheless, in both groups the majority (65.2% and 71.0%, respectively) responded with “I don’t know.” The correct answer, based on our previous critical reviews [11], would have been that such studies are not reliable; however, very few respondents selected this option. This is understandable, given that our earlier publications [11,28] remain the only work to date addressing the methodological confounders of ketogenic diet clinical trials and their implications.
(iv) Similar trends were observed regarding willingness to try ketogenic diet. Students who stated they would consider ketogenic diet for weight loss or health reasons were more likely to view the research as reliable (29.4%) compared to those who would not (17.3%). Among academic staff, a similar pattern emerged (37% vs. 10.7%).

### 4.7. Question 6: “If You Wanted to Learn More About Ketogenic Diets, Which of These Resources Would You Use?”

This question was of particular interest given that, ideally, medical databases should provide the most reliable information. Unfortunately, in the case of ketogenic diets, this is not true [11,28]. The second most appropriate option would be consulting a healthcare professional, if they themselves are adequately informed.

In our study, many students (64%) indicated that they would turn to healthcare professionals for information, whereas academic staff most frequently selected PubMed (64%) as their primary source (Figure 4 and Figure 5). In contrast, Almarshedy et al. [33] reported that most participants obtained their knowledge of ketogenic diet from the internet, while Altamimi et al. [35] found that respondents considered social media to be the most reliable source of information.

### 4.8. Limitations

This study has several limitations that should be acknowledged.

The overall response rate was relatively low (3.32% among students and 6.29% among academic staff), which increases the likelihood of non-response bias, as individuals with greater interest in the topic may have been more inclined to participate.

The representativeness of the sample could not be determined, since detailed demographic data on the university’s student and staff population (e.g., by age, sex, faculty, or program) are not publicly available. Moreover, the voluntary, self-selection, non-probability sampling design further limits representativeness and raises the possibility of self-selection bias.

Data was self-reported, which may be subject to recall bias or social desirability bias, particularly regarding weight, height, and dietary habits. Additionally, while the questionnaire was carefully developed and piloted, it was self-designed after all and has not been formally validated through psychometric methods. Therefore, comparability with other surveys and the reliability of the knowledge score should be interpreted with caution. Additionally, the scoring system included both positive and negative points, (which was intended to reflect the quality of knowledge) was based on researcher judgment and may have introduced further bias.

The cross-sectional design limits the ability to draw causal inferences, and the results cannot be generalized beyond the University of Pécs. Furthermore, interpretation of knowledge scores is complicated by the fact that much of the scientific literature on ketogenic diets is itself methodologically limited or inconsistent, which makes it difficult to distinguish between gaps in participants’ knowledge and/or shortcomings of the available evidence.

## 5. Conclusions

This study is to our knowledge, the first questionnaire-based investigation of ketogenic diets that not only assessed knowledge and perceptions, but also explicitly addressed the methodological shortcomings and misinterpretations present in the current scientific literature. Using a self-developed and piloted questionnaire among students and academic staff at the University of Pécs, we found that overall knowledge about ketogenic diets was low, with only 7.3% of students and 13.5% of staff achieving at least 60% of the maximum knowledge score. Contrary to our hypothesis, healthcare-related faculties did not consistently outperform non-healthcare faculties; in fact, students from unrelated disciplines occasionally achieved higher scores, highlighting an opportunity to reconsider assumptions about informal learning and the sources through which students acquire nutrition-related knowledge.

Completion of nutrition-related courses and reliance on scientific information sources such as PubMed were associated with higher knowledge scores, while current adherence to ketogenic diets among staff was negatively associated with knowledge. Importantly, many participants expressed uncertainty about the reliability of ketogenic research, which is consistent with our earlier reviews demonstrating that existing clinical studies and meta-analyses in this field are methodologically compromised. These results highlight the need for critical appraisal, even when interpreting information from scientific literature.

This limited level of awareness is concerning, as effective prevention of cardiovascular and oncological diseases cannot be achieved without broader public understanding of such fundamental nutrition concepts. Healthcare professionals working in this area should also be aware of the physiological and pathophysiological processes that contraindicate the recommendation of ketogenic diets, particularly given the existence of numerous other well-established diets that provide proven long-term health benefits without extreme macronutrient imbalances. As we have emphasized throughout this work, ketogenic diets may appear advantageous for disease treatment but not taking the whole person into consideration. This leads to a reductionist perspective that risks overlooking broader aspects of health.

Taken together, our findings support the urgent need for critical appraisal skills and reliable evidence-based nutrition education to be strengthened not only among healthcare professionals, but also across the broader population. A more comprehensive public understanding of fundamental nutrition concepts is essential for effective disease prevention and for counteracting misinformation surrounding dietary practices such as the ketogenic diet.

The findings of this study underscore the need for further research aimed at bridging the gap between scientific evidence and public understanding of nutrition-related topics. Future investigations should expand this questionnaire-based approach to include participants from other universities and diverse cultural contexts, enabling cross-national comparisons of knowledge and perceptions regarding ketogenic diets and other dietary interventions. Longitudinal studies could also help assess whether evidence-based nutrition education programs can improve critical appraisal skills and reduce the spread of dietary misinformation among both students and the general population.

For the scientific community, our results emphasize the importance of addressing methodological transparency in ketogenic diet research and of developing validated tools to measure nutrition literacy more broadly. For the general population, this study highlights the urgent need for accessible, reliable, and evidence-based nutrition education. Promoting critical thinking about dietary trends may ultimately contribute to more informed health choices and improved disease prevention strategies at the population level.

## Figures and Tables

**Table 1:** Demographic characteristics of the students and academic staff.

		Students		Academic Teachers	
Sex		*n*	%	*n*	%
	Male	207	29.2%	47	38.2%
	Female	498	70.1%	75	61.0%
	Prefer not to answer	3	0.4%	-	-
	Other	2	0.3%	1	0.8%
	Total	710	100.0	123	100.0
Age		*n* = 708	Age	*n* = 123	Age
	Mean		26.5		45.8
	SD		9.7		10.8
	Median		22.0		45.0
Faculty		*n*	%	*n*	%
	Law	40	5.6	1	0.8
	Medical School	236	33.2	63	51.6
	Humanities and Social Sciences	119	16.8	10	8.2
	Health Sciences	139	19.6	13	10.7
	Pharmacy	17	2.4	5	4.1
	Business and Economics	41	5.8	6	4.9
	Cultural Sciences, Education and Regional Development	13	1.8	2	1.6
	Engineering and Information Technology	50	7.0	6	4.9
	Music and Visual Arts	11	1.5	7	5.7
	Sciences	44	6.2	9	7.4
	Total	710	100.0	122	99.2
University Program		*n*	%	*n*	%
	1st Year	277	39.1		
	2nd Year	159	22.5		
	3rd Year	113	16.0		
	4th Year	70	9.9		
	5th Year	17	2.4		
	6th Year	15	2.1		
	PhD student	46	6.5		
	Other	8	1.1		
	Total	708	99.7		
BMI		*n*	%	*n*	%
	18.5–24.9	509	72.0	64	52.0
	25-	198	28.0	59	48.0
	Total	707	99.6	123	100.0

**Table 2:** Calculation method of the knowledge score related to ketogenic diets.

Questions	Answers	Knowledge Score
What do you think are the characteristic features of ketogenic diets? (Select all that apply)	I don’t know	0 point
	Mostly animal-based fats should be consumed.	0 point
	Mostly plant-based fats should be consumed.	0 point
	One’s fat intake should include both animal and plant-based fats.	0 point
	40% of one’s daily food intake should consist of proteins.	−1 point
	Simple carbohydrates (e.g., cakes, chocolate) cannot be consumed, but fruits are allowed.	0 point
	More than half of the diet consists of vegetables.	−1 point
	The diet improves cholesterol levels.	0 point
	The diet improves blood sugar levels.	0 point
	The diet is proven effective for weight loss.	0 point
	One’s carbohydrate intake is so low that it can lead to dizziness, headaches, and difficulty concentrating.	1 point
	In the long run, the diet can lead to kidney and liver problems.	1 point
	The diet can cause constipation.	1 point
	The diet can cause diarrhea.	1 point
	The diet can induce a metabolic state similar to starvation without needing to reduce one’s calorie intake.	1 point
	While adhering to the diet, ketone bodies are produced from carbohydrates.	−1 point
	Ketogenic Diets are proven to not be harmful to one’s health.	0 point
Please list as many types of foods that typically contain carbohydrates as you can.	Fruits, vegetables, grains etc.	1 point
	All other versions	0 point
In your opinion, what characterizes “nutritional ketosis”. (Select all that apply)	I don’t know	0 point
	Any amount of ketone bodies in the blood.	−1 point
	Ketone body levels in the blood between 0.5–3.0 mmol/L.	1 point
	Ketone body levels in the blood between 10–20 mmol/L.	−1 point
	This is a normal/physiological state.	1 point
	This is a pathological state.	−1 point
	During Nutritional Ketosis, the brain and other organs have sufficient energy.	1 point
What do you think is an indicator that someone on a ketogenic diet has reached nutritional ketosis?	I don’t know	0 point
	Other (Measuring the presence of ketone bodies in the blood, urine)	1 point
In your opinion, how many calories are consumed per day on a ketogenic diet?	I don’t know	0 point
	Approximately 2000 kcal	1 point
	More than a typical diet (which is ~2000 kcal for a sedentary lifestyle)	0 point
	About 600–800 kcal	0 point
	About 1500 kcal	0 point
Do you think it is possible to lose weight on a diet that follows any macronutrient ratio (carbohydrate:fat:protein)?	I don’t know	0 point
	Yes	1 point
	No	0 point
	If yes, in what instances? (caloric deficit)	1 point
In your opinion, is a sufficiently low-calorie intake enough for weight loss?	I don’t know.	0 point
	Yes	1 point
	No	0 point
When following a Ketogenic Diet, what can one eat?	I don’t know	0 point
	Mostly fat, a small amount of protein, very small amount of carbohydrates	1 point
	30% of energy must come from fats, 15% from protein, and 55% from carbohydrates	0 point
	1/3 fat intake, 1/3 protein intake, 1/3 carbohydrates intake	0 point
In your opinion, are animal-derived fats or plant-derived fats healthier?	I don’t know	0 point
	Animal-derived fats are healthier.	0 point
	Plant-derived fats are healthier.	1 point
	Both are unhealthy.	0 point
	Both are equally healthy and necessary for maintaining health.	0 point
Which types of fatty acids are mainly found in animal-derived foods?	I don’t know	0 point
	Mainly saturated fatty acids	1 point
	Mainly omega-6 fatty acids	0 point
	Mainly omega-3 fatty acids	0 point
	Mainly mono-unsaturated fatty acids	0 point

**Table 3:** Comparison of knowledge scores by participant characteristics.

Variable	Group	Statistical Test	*p*-Value	Significant Pairwise Difference (Post Hoc)	Mean Difference (Points)
**Sex (students)**	Male vs. Female	Mann–Whitney U	0.892	–	–
**Sex (academic staff)**	Male vs. Female	Mann–Whitney U	0.327	–	–
**Faculty (all students combined)**	10 faculties	One-way ANOVA (Dunnett’s T3)	0.017	Medical School > Humanities & Social Sciences	1.231
**Faculty (Hungarian students)**	10 faculties	One-way ANOVA (Dunnett’s T3)	0.001	Med > Law (*p* = 0.022); Med > Hum&Soc (*p* = 0.007); Eng&IT > Law (*p* = 0.003); Eng&IT > Hum&Soc (*p* = 0.001)	1.75–2.63
**Faculty (English-program students)**	10 faculties	One-way ANOVA	0.384	n.s.	–
**Faculty (academic staff)**	10 faculties	One-way ANOVA	0.435	n.s.	–
**Correlation between correct and incorrect answers (students)**	–	Pearson r	0.115	*p* = 0.010	Weak positive
**Association: reliability of research vs. knowledge adequacy (students)**	3 response categories	χ^2^ test	0.004	“Unreliable” > “Unable to judge”	–

Eng&IT = Faculty of Engineering and Information Technology; Hum&Soc = Faculty of Humanities and Social Sciences; χ^2^ test = chi-square test; Law = Faculty of Law; Med = Medical School; n.s. = not significant.

**Table 4:** Logistic regression predictors of adequate knowledge (≥ 60% of maximum score).

Variable	Group	Odds Ratio (OR)	95% CI	*p*-Value	Direction of Association
Completed nutrition-related course	Students	4.701	1.915–11.537	0.001	↑ Adequate knowledge
Information source: Internet	Students	2.615	1.233–5.545	0.012	↑ Adequate knowledge
Information source: PubMed	Students	4.475	1.957–10.231	<0.001	↑ Adequate knowledge
Current adherence to KD	Academic staff	0.430	0.221–0.834	0.013	↓ Adequate knowledge

↑ Increase; ↓ decrease.

**Table 5:** Key questionnaire items and response patterns.

Question/Item	Correct Response (%)	Notes/Interpretation
**“In your opinion, are the results of scientific research on ketogenic diets reliable?”**	*p* = 0.004 (students); *p* = 0.060 (staff)	Students who considered research “unreliable” more often achieved adequate knowledge (17.8%) than those who considered it “reliable” (10.3%) or were “unable to judge” (5.1%).
**List foods that typically contain carbohydrates**	27.3% (Hungarian); 18.6% (English)	Highest scores awarded for naming both fruits and vegetables.
**What characterizes nutritional ketosis?**	5.8% (all participants); 5.9% (students); 5.4% (staff); 8.8% (Medical School students)	Few participants identified all three correct characteristics.
**Is a sufficiently low-calorie intake enough for weight loss?**	49.2% answered “yes”	Correctly reflects the law of energy conservation.
**When following a ketogenic diet, what can one eat?**	68% correct overall (students 67.5%, staff 70.3%)	Most participants identified the correct food types.
**Which type of fats are healthier (animal vs. plant)?**	28.2% correctly identified plant-derived fats as healthier	Indicates uncertainty regarding dietary fat quality.
**Which fatty acids are mainly found in animal-derived foods?**	53.1% correctly answered “saturated fatty acids”	Basic biochemical knowledge moderately represented.

**Table 6:** Factors associated with having heard of the ketogenic diet (Question 1).

Variable	Group	Odds Ratio (OR)	95% CI	*p*-Value	Interpretation
**Sex (female)**	Students	1.456	1.012–2.097	0.043	Females were more likely to have heard of KD.
**Age (per year)**	Students	1.030	1.008–1.053	0.007	Older students had higher awareness.
**Year of study**	Students	1.201	1.082–1.335	0.001	Awareness increased with study year.
**Language (English vs. Hungarian)**	Students	0.558	0.389–0.800	0.002	English-program students were less likely to have heard of KD.
**Completed nutrition-related course (no)**	Students	0.208	0.072–0.596	0.003	Lack of nutrition coursework reduced awareness.
**Language (English vs. Hungarian)**	Academic staff	0.046	0.004–0.551	0.015	Staff completing the English version were less likely to have heard of KD.

CI = Confidence Interval; KD = Ketogenic Diet; OR > 1 indicates higher odds of awareness; OR < 1 indicates lower odds. Only statistically significant predictors are shown (*p* < 0.05).

**Table 7:** Factors associated with currently or previously following a ketogenic diet (Question 2).

Variable	Group	Odds Ratio (OR)	95% Confidence Interval (CI)	*p*-Value	Interpretation
**Healthcare professional recommendation (yes)**	Students	4.375	1.874–10.212	0.001	Students who received professional advice were over four times more likely to follow a KD.
**Age (per year)**	Students	1.044	1.023–1.066	<0.001	Older students were more likely to currently or previously follow a KD.
—	Academic staff	—	—	—	No significant associations identified.

CI = Confidence Interval; KD = Ketogenic Diet; OR > 1 indicates increased odds of KD adherence; only statistically significant predictors are shown (*p* < 0.05).

**Table 8:** Factors associated with willingness to follow a ketogenic diet for weight loss or health reasons (Question 3).

Variable	Group	Odds Ratio (OR)	95% Confidence Interval (CI)	*p*-Value	Interpretation
**Sex (female)**	Students	0.549	0.362–0.832	0.005	Female students were significantly less likely to express willingness to follow a KD.
**Age (per year)**	Students	1.036	1.017–1.057	<0.001	Older students were more likely to consider following a KD.
—	Academic staff	—	—	—	No significant associations identified.

CI = Confidence Interval; KD = Ketogenic Diet; OR > 1 indicates higher odds of willingness to follow a KD; only statistically significant predictors are shown (*p* < 0.05).

**Table 9:** Summary of responses to Question 4.

Comparison/Variable	Group 1	Group 2	Proportion Reporting ‘Yes’ (%)	Statistical Test	*p*-Value	Interpretation
**Student subsamples**	Hungarian	English	5.2% vs. 4.3%	χ^2^ test	0.861	No significant difference between language groups.
**BMI category**	Non-overweight	Overweight	3.7% vs. 7.9%	χ^2^ test	0.044	Overweight students were significantly more likely to receive such a recommendation.
**Followed or tried KD**	Recommendation = Yes	Recommendation = No	52.0% vs. 17.1%	χ^2^ test	<0.001	Those advised by professionals were more likely to have tried or be following a KD.
**Academic staff**	—	—	0% reported “Yes”	—	—	No staff member reported receiving a recommendation.

KD = Ketogenic Diet; χ^2^ test = chi-square test; statistical significance was set at *p* < 0.05.

**Table 10:** Associations between respondents’ perceptions of the reliability of ketogenic diet research (Question 5) and selected variables.

Variable	Group(s) Compared/Key Finding	Statistical Test	*p*-Value	Interpretation
**Faculty**	Distribution of responses differed significantly across faculties; 38.5% of Pharmacy students considered KD research reliable.	χ^2^ test	0.035	Faculty affiliation significantly influenced perceived reliability.
**Field of study**	42.9% of Dietetics students judged KD research reliable.	χ^2^ test	0.020	Field of study was significantly associated with perceived reliability.
**Year of study**	—	χ^2^ test	n.s.	No significant association observed.
**Current/past KD adherence**	Among KD followers: 31.6% reliable vs. 3.2% unreliable vs. 65.2% “I don’t know.” Non-followers: 18.8% reliable vs. 10.2% unreliable.	χ^2^ test	0.005	Students who had tried KD were more likely to consider KD research reliable.
**Willingness to follow KD**	Willing: 29.4% reliable vs. 3.1% unreliable; Unwilling: 17.3% reliable vs. 11.7% unreliable.	χ^2^ test	<0.001	Willingness to follow KD strongly associated with higher trust in KD research.
**Academic staff—willingness**	Willing staff: 37% reliable vs. 0% unreliable; Unwilling: 10.7% reliable vs. 14.3% unreliable.	χ^2^ test	0.001	Similar pattern observed among staff; willingness correlated with higher perceived reliability.

KD = Ketogenic Diet; χ^2^ test = chi-square test; n.s. = non-significant (*p* > 0.05); significance threshold = *p* < 0.05. Percentages reflect the proportion selecting each response category within subgroups.

## Data Availability

The raw data supporting the conclusions of this article will be made available by the authors on request due privacy.

## Supplementary Materials

The following supporting information can be downloaded at: https://www.mdpi.com/article/10.3390/nu17213327/s1, Table S1: Questionnaire; Table S2: Demographic data: Nationality.

## Abbreviations

The following abbreviations are used in this manuscript:

ANOVA	Analysis of Variance
BMI	Body Mass Index
CI95%	95% Confidence Interval
Eng&IT	Faculty of Engineering and Information Technology
Hum&Soc	Faculty of Humanities and Social Sciences
KD	Ketogenic Diet
χ^2^ test	Chi-square Test
Med	Medical School
n.s.	Non-significant
OR	Odds Ratio
SEM	Standard Error of Mean
SPSS	Statistical Package for the Social Sciences
