# Supplementary material for: Opinions on Ketogenic Diets Among Students and Academic Teachers at the University of Pécs, Hungary: A Cross-Sectional Survey
Source: Nutrients. 2025 Oct 22;17(21):3327. doi: 10.3390/nu17213327 (PMC12610887; doi:10.3390/nu17213327)
Supplement: Supplementary file 1 [file nutrients-17-03327-s001.zip › Table S1. Ketogenic Diet Questionnaire.pdf]

Opinions on ketogenic diets among instructors and students at the University of Pécs

Dear Participant!

Trying to maintain a "healthy lifestyle" has become an integral part of many people's daily life. The University of Pécs Medical School researchers from the Department of Public Health Medicine, are interested in the opinions of University of Pécs students and instructors regarding Ketogenic Diets via this questionnaire.
The questions in the first part of the questionnaire are designed to ask your personal characteristics, including demographic and lifestyle information. The questions in the second part will help us to gather your opinions on ketogenic diets. Answering these questions does not require any particular expertise or special knowledge, as anyone can have their own experiences or opinions about ketogenic diets.
There are no right or wrong answers to any of the questions; the aim of the research is solely to gather individuals' opinions. Participation in the survey is completely voluntary and anonymous. The data collected from this questionnaire will be used solely for research purposes and will not be shared with any third parties under any circumstances. Any findings presented will be in an aggregated and generalized format. We kindly ask you for your help by completing this online 31 question questionnaire which should take approximately 15 minutes. In order to ensure the accuracy of the data, it is necessary to answer every question. However, you can stop completing the questionnaire at any time without explanation.

Thank you in advance for your response!

General Questions Related to Demographic Data

1. Please indicate your sex.
Male
Female
Other / Prefer not to answer.

2. Please indicate your **year** of birth.
....

3. Please indicate your **nationality**.
....

4. At the University of Pécs I am a ...
Student

Teacher / Professor
I have graduated (e.g., Physician, Biologist, Chemist, Pharmacist, Dietitian, etc.)
Other ....

5. At the University of Pécs, I study and/or teach in the ... (Select all that apply)
Faculty of Law
Medical School
Faculty of Humanities and Social Sciences
Faculty of Health Sciences
Faculty of Pharmacy
Faculty of Business and Economics
Faculty of Cultural Sciences, Education and Regional Development
Faculty of Engineering and Information Technology
Faculty of Music and Visual Arts
Faculty of Sciences

6. Within this, what field(s) do you teach or study within?
....

7. My workplace is ... (e.g., clinic/institute):
......

8. If you are a current student, what year of your program are you in?
I am not a current student.
1st Year
2nd Year
3rd Year
4th Year
5th Year
6th Year
Other: ...

9. Are you currently taking or have you completed any nutrition-related training or courses?
Yes
No
If yes, please indicate the name of the educational institution and training or course that you are currently taking or completed: ...

10. Please indicate your height in cm.
...

11. Please indicate your weight in kg.
...

12. How many times a week do you attend the gym?
I do not attend the gym.
I used to attend the gym regularly for several years, but I am not currently attending.

1 - 2 times a week
3 - 4 times a week
5 times or more a week
Other: ...

13. What type of exercise(s) do you perform at the gym?
Weightlifting, Strength-based and/or machine-based exercises
Aerobic, “Cardio”, High Intensity Interval Training (HIIT), and/or Circuit Training
Both
Other: ...

14. If you are not currently attending the gym, do you partake in any other type of exercise(s) regularly?
Yes
No
If yes, what kind (e.g., Yoga, Pilates, Sport, Outdoor Activities, etc.)? ...

General Questions Related to Ketogenic Diet(s)

15. Have you heard of Ketogenic Diet(s) before?
Yes
No
If the answer is no – the questionnaire ends here.

16. Are you currently following a Ketogenic Diet?
Yes
No, but in the past I followed a Ketogenic Diet
No

17. Would you follow a Ketogenic Diet for the purpose of losing weight, or being “more healthy”?
Yes
No
If yes, why? ...
If no, why not? ...

18. Has a healthcare professional ever suggested you follow a Ketogenic Diet?
Yes
No
If yes, for what reason? ...

19. In your opinion, are the results of scientific research done on ketogenic diets reliable?
Yes
No
I don’t know
If yes, why? ...

If no, why not? ...

Questions to Assess One's Opinion About Ketogenic Diets

If you are unsure about the answer to any of the following questions, we kindly ask you to please choose the "I don't know" option. There is no disadvantage to selecting this option, but it is an important factor in our research.

20. What do you think are the characteristic features of ketogenic diets? (Select all that apply)
I don't know.
Mostly animal-based fats should be consumed.
Mostly plant-based fats should be consumed.
One's fat intake should include both animal and plant-based fats.
40% of one's daily food intake should consist of proteins.
Simple carbohydrates (e.g., cakes, chocolate) cannot be consumed, but fruits are allowed.
More than half of the diet consists of vegetables.
The diet improves cholesterol levels.
The diet improves blood sugar levels.
The diet is proven effective for weight loss.
One's carbohydrate intake is so low that it can lead to dizziness, headaches, and difficulty concentrating.
In the long run, the diet can lead to kidney and liver problems.
The diet can cause constipation.
The diet can cause diarrhea.
The diet can induce a metabolic state similar to starvation without needing to reduce one's calorie intake.
While adhering to the diet, ketone bodies are produced from carbohydrates.
Ketogenic Diets are proven to not be harmful to one's health.
Other: ...

21. Please list as many types of foods that typically contain carbohydrates as you can.
....

22. In your opinion, what characterizes "nutritional ketosis" (Select all that apply)
I don't know.
Any amount of ketone bodies in the blood.
Ketone body levels in the blood between 0.5 - 3.0 mmol/L.
Ketone body levels in the blood between 10 - 20 mmol/L.
This is a normal/physiological state.
This is a pathological state.
During Nutritional Ketosis, the brain and other organs have sufficient energy.
Other: ...

23. What do you think is an indicator that someone on a ketogenic diet has reached nutritional ketosis?
....
I don't know.

24. In your opinion, how many calories are consumed **per day** on a ketogenic diet? (Select all that apply)
I don't know.
Approximately 2000 kcal
More than a typical diet (which is ~2000 kcal for a sedentary lifestyle)
About 600 kcal
About 1500 kcal
Other: ...

25. Do you think it is possible to lose weight on a diet that follows **any** macronutrient ratio (carbohydrate–fat–protein)? *(According to WHO, the normal macronutrient ratio in terms of energy percentage is carbohydrate: 55-75%, fat: 15-30%, protein: 10-15%)*
I don't know.
Yes
No
If yes, in what instances? ...
If no, why not? ...

26. In your opinion, is a sufficiently low-calorie intake enough for weight loss?
I don't know.
Yes
No
If no, why not? ...

27. When following a Ketogenic Diet, what **can** one eat?
I don't know.
Mostly fat, a small amount of protein, very small amount of carbohydrates
30% of energy must come from fats, 15% from protein, and 55% from carbohydrates
1/3 fat intake, 1/3 protein intake, 1/3 carbohydrates intake

28. In your opinion, are animal-derived fats or plant-derived fats healthier?
I don't know.
Animal-derived fats are healthier.
Plant-derived fats are healthier.
Both are unhealthy.
Both are equally healthy and necessary for maintaining health.
Other: ...

29. Which types of fatty acids are mainly found in animal-derived foods?
I don't know.
Mainly saturated fatty acids
Mainly omega-6 fatty acids
Mainly omega-3 fatty acids
Mainly mono-unsaturated fatty acids
Other: ...

30. If you wanted to learn more about Ketogenic Diets, which of these resources would you use? (Select all that apply)
I don't know.
Healthcare Professional
Facebook / Social Media
Internet (e.g., Google)
PubMed / Other Scientific Databases
Other: ...

31. In your opinion, is the following statement True or False. *"The effects of ketogenic diet types are adequately supported by scientific evidence."*
True
False
I don’t know.

If you have any comments or feedback regarding the questionnaire, please feel free to write them here.
....

Thank you for your answers!
