# Supplementary material for: Opinions on Ketogenic Diets Among Students and Academic Teachers at the University of Pécs, Hungary: A Cross-Sectional Survey
Source: Nutrients. 2025 Oct 22;17(21):3327. doi: 10.3390/nu17213327 (PMC12610887; doi:10.3390/nu17213327)
Supplement: Supplementary file 1 [file nutrients-17-03327-s001.zip › Table S2. Nationality.pdf]

		Students		Academic teachers	
Nationality		n	%	n	%
	Hungarian	461	65.2	117	95.1
	Alban	1	0.1		
	Algeria	2	0.3		
	Angola	1	0.1		
	Azerbaijan	2	0.3		
	Bangladesh	4	0.6		
	Belarus	2	0.3		
	Belgium	1	0.1		
	Bosnia	1	0.1		
	Botswana	1	0.1		
	Brazil	5	0.7	1	0.8
	GB	1	0.1		
	Kameron	1	0.1		
	Canada	2	0.3		
	China	6	0.8		
	Colombia	1	0.1		
	Cyprus	1	0.1		
	Germany	24	3.4		
	Ecuador	1	0.1		
	Ethiopia	1	0.1		
	Philippines	1	0.1		
	Finnish	1	0.1		
	French	3	0.4		
	Greek	1	0.1		
	Guatemala	1	0.1		
	Indian	17	2.4	1	0.8
	Indonesia	3	0.4		
	Iran	19	2.7		
	Irak	3	0.4		
	Japan	4	0.6		
	Jordan	8	1.1		
	Kenya	8	1.1		
	South-Korea	4	0.6		
	Kosovo	1	0.1		
	Macedonia	1	0.1		
	Maldives	1	0.1		
	Moldova	3	0.4		
	Mongolia	2	0.3		
	Morocco	1	0.1		
	Myanmar	2	0.3		
	Nigeria	11	1.6		
	Norwegian	28	4.0		
	Pakistan	9	1.3		
	Romanian	7	1.0	1	0.8
	Russia	3	0.4		
	Sierra-Leone	4	0.6		
	Spain	1	0.1		
	Syrian	3	0.4	1	0.8
	Tanzania	1	0.1		
	Tunesia	1	0.1		

Turkish	6	0.8		
USA	1	0.1		
Uzbekistan	2	0.3		
Vietnam	3	0.4		
Zambia	1	0.1		
Zimbabwe	2	0.3		
USA-Pakistan	1	0.1		
USA-Hungarian	1	0.1	1	0.8
USA-Norwegian	1	0.1		
German-Hungarian	1	0.1		
German-USA	1	0.1		
Hungarian-Russian	1	0.1		
Indian-Philippines	1	0.1		
Romanian-Hungarian	5	0.7		
Slovak	3	0.4		
Serbian-Hungarian	1	0.1	1	0.8
Hungarian-Israel	1	0.1		
Hungarian-Ukrainian	1	0.1		
Hungarian-Norwegian	1	0.1		
Hungarian-Turkish	1	0.1		
Chad	1	0.1		
Total	707	99.6	123	100.0
